# Supplementary material for: Decoding attention control and selection in visual spatial attention
Source: Hum Brain Mapp. 2020 Jun 16;41(14):3900–21. doi: 10.1002/hbm.25094 (PMC7469865; doi:10.1002/hbm.25094)
Supplement: Supplementary file 1 — Appendix S1. Supporting Information. [file HBM-41-3900-s001.docx]

# Decoding Attention Control and Selection in Visual Spatial Attention

***Supplemental Methods***

**Cue-related alpha power analysis**

The alpha power of cue-related EEG epochs was computed using a standard approach of temporal spectral evolution (Hong et al., 2015; Rihs et al., 2009; Thut et al., 2006; Worden et al., 2000). Briefly, each cue-related EEG epoch was filtered into alpha (8-13 Hz) band, rectified and then smoothed by averaging within a moving time window (width: 100 ms), yielding alpha power at each time point and each electrode for each cue-related epoch.

**Cue-related alpha-based decoding analysis**

The decoding procedure was identical to that for the cue-related ERP-based decoding, except that the decoding of attention condition (cue left vs. cue right) was based on the spatial distribution of alpha power over the scalp.

***Supplemental Results***

**
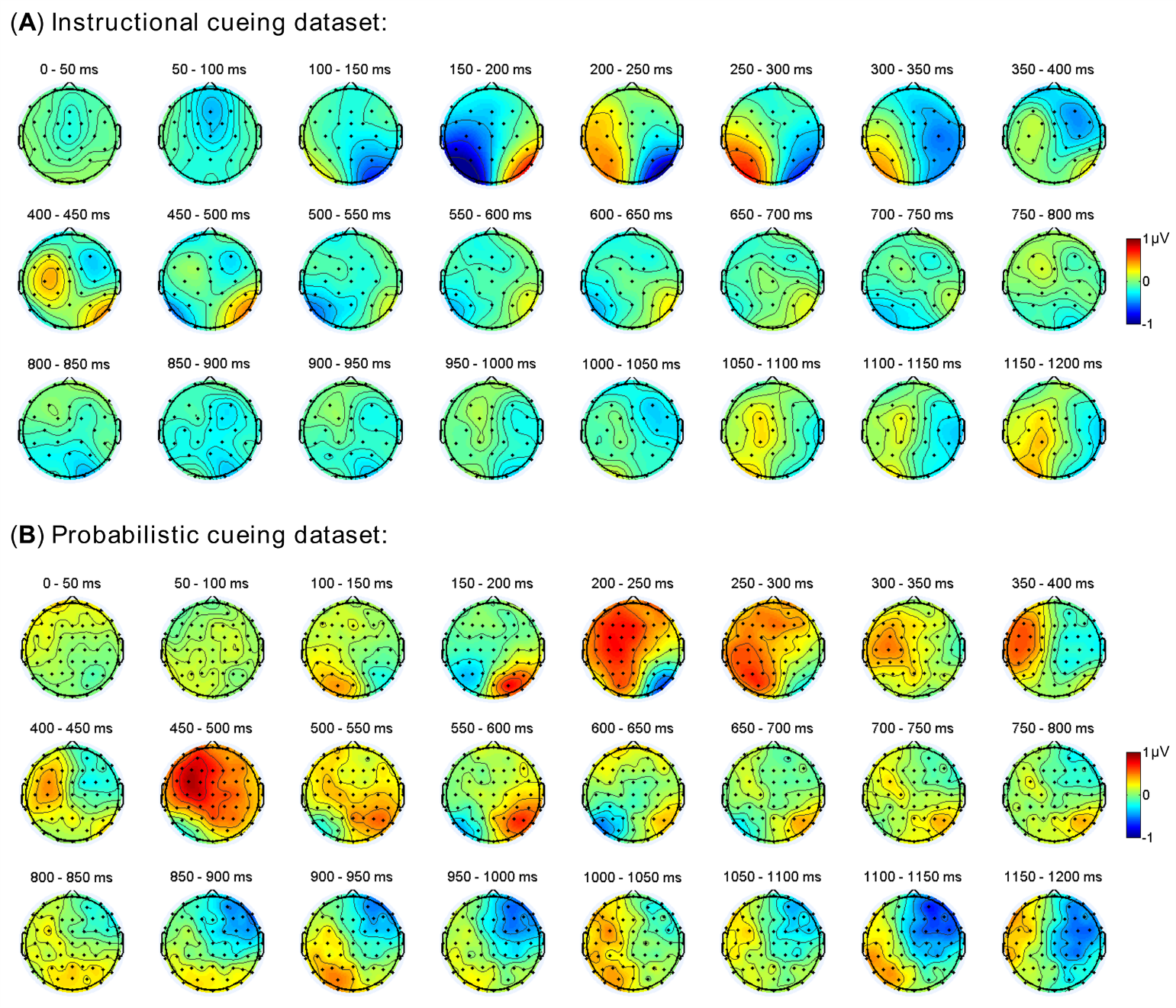
**

**Figure S1.** Topographical maps of ERP difference waves (cue left *minus* cue right) from successive time points within the indicated windows were averaged and shown for the two experiments (*A*: instructional cueing; *B*: probabilistic cueing).


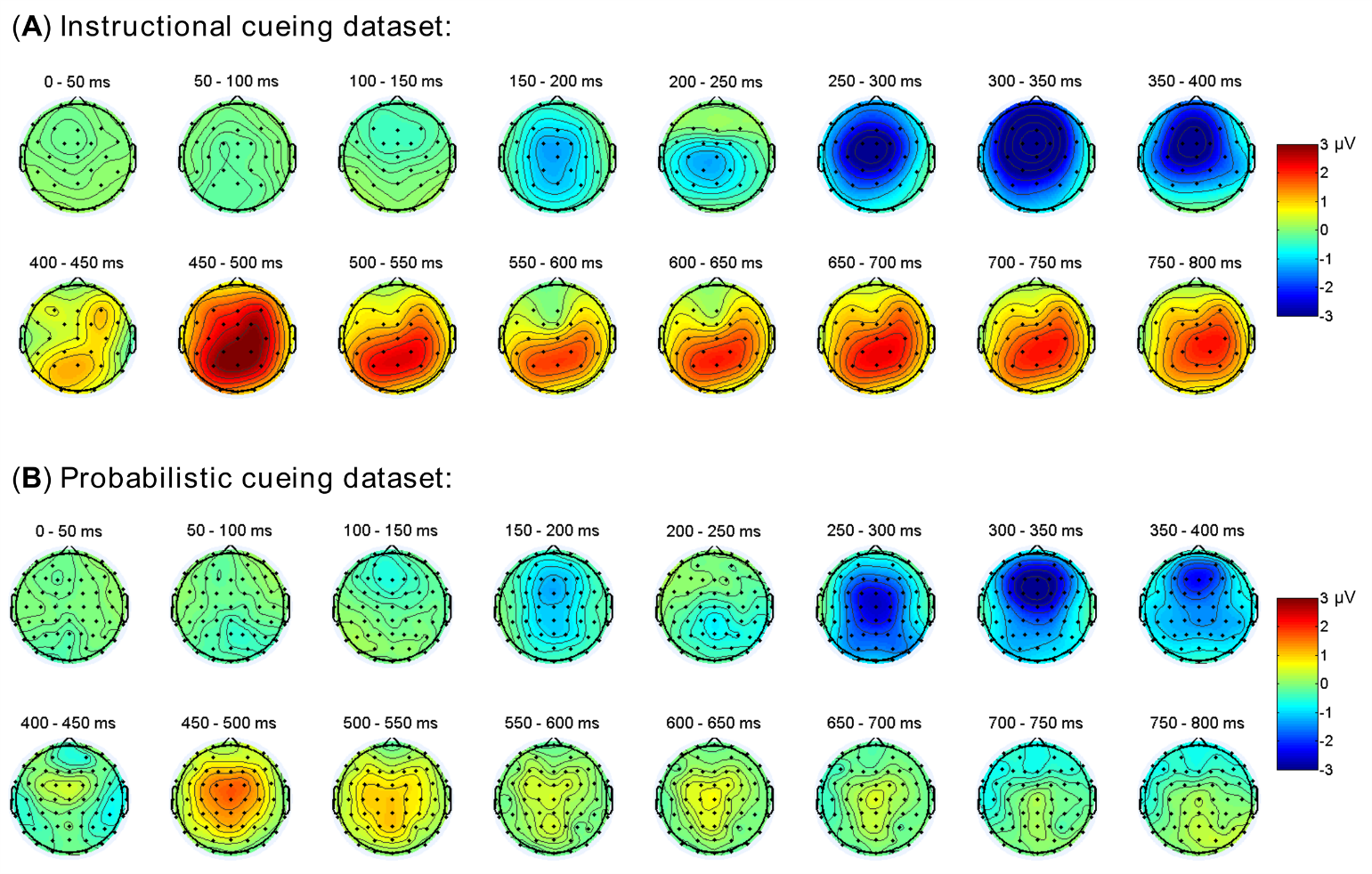


**Figure S2.** Topographical maps of ERP difference waves (cued target *minus* uncued target, with left and right targets combined) from successive time points within the indicated windows were averaged and shown for the two experiments (*A*: instructional cueing; *B*: probabilistic cueing).


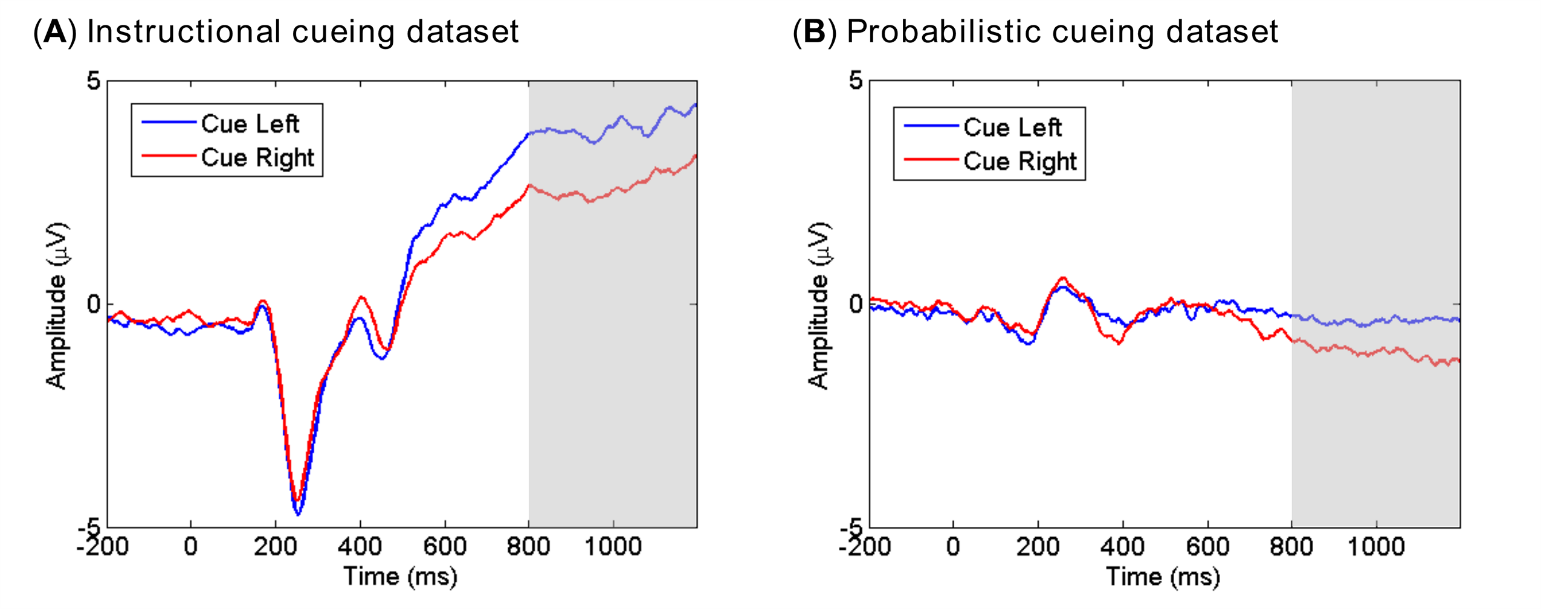


**Figure S3**. Grand-averaged HEOG during cue-target interval for the two experiments. The HEOG in (*A*) was recorded by one electrode placed on the outer ocular canthus of the left eye, and the HEOG in (*B*) was derived from the voltage difference between FT9 and FT10 (FT9 *minus* FT10). The HEOG difference between cue left and cue right during 800-1200 ms interval (grey region) is significantly different from 0 in (*A*) (1.32 ± 0.31 μV, *t*_(29)_ = 4.219, *p* < 0.001, Cohen’s d = 0.770), but only marginally different from 0 in (*B*) (0.71 ± 0.41 μV, *t*_(25)_ = 1.744, *p* = 0.093, Cohen’s d = 0.342).


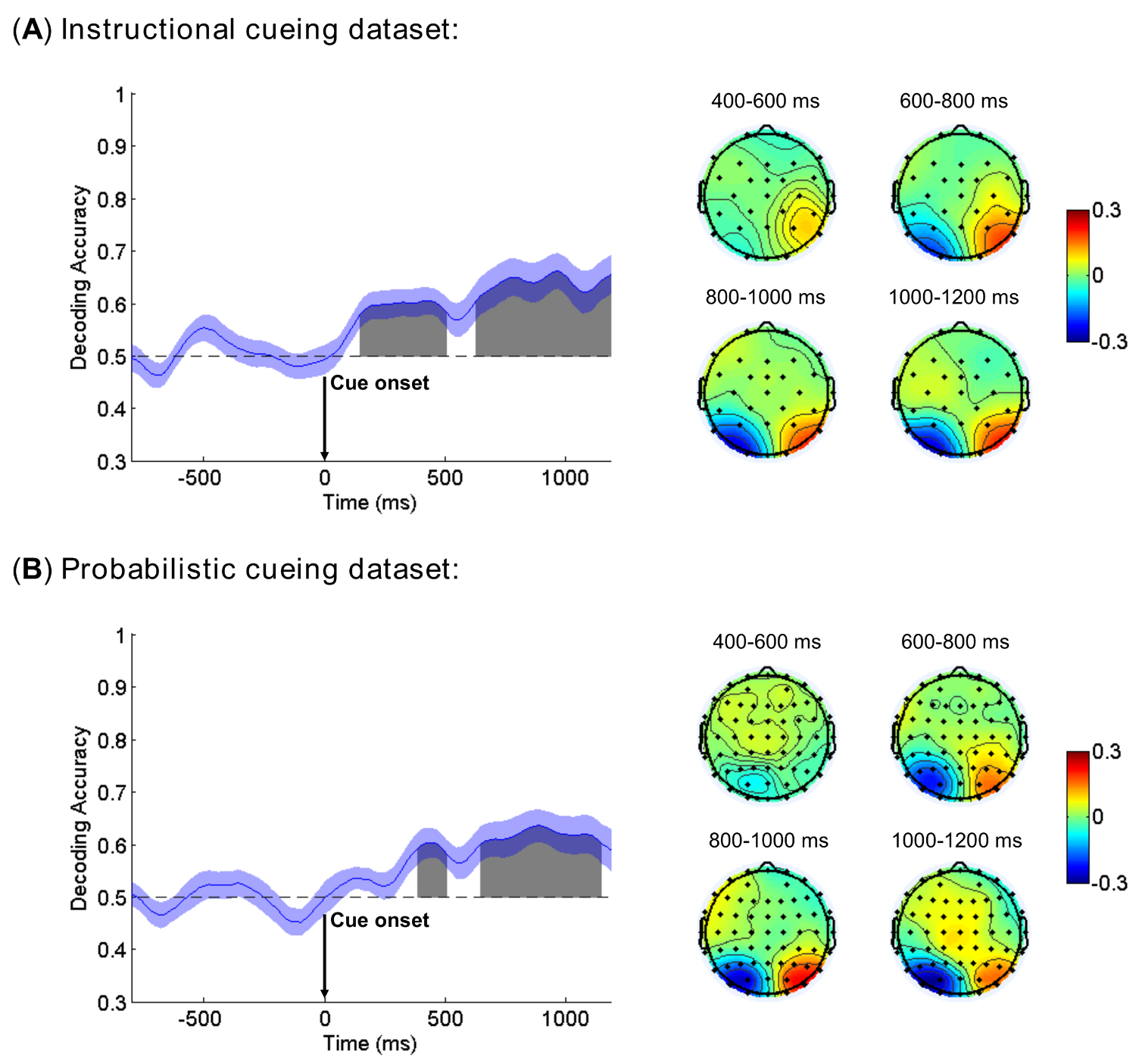


**Figure S4**. Mean accuracy of alpha-based multivariate decoding for cue-related neural processing (cue left vs. cue right) in the Instructional cueing dataset (*A*) and Probabilistic cueing dataset (*B*). Chance level performance (0.5) is indicated by the horizontal dash lines. Gray areas indicate clusters of time points in which the decoding was significantly greater than chance after the FDR correction for multiple comparisons. The blue shading indicates ±1 SEM. Weight maps from successive time points within the indicated windows were averaged and shown on the right.


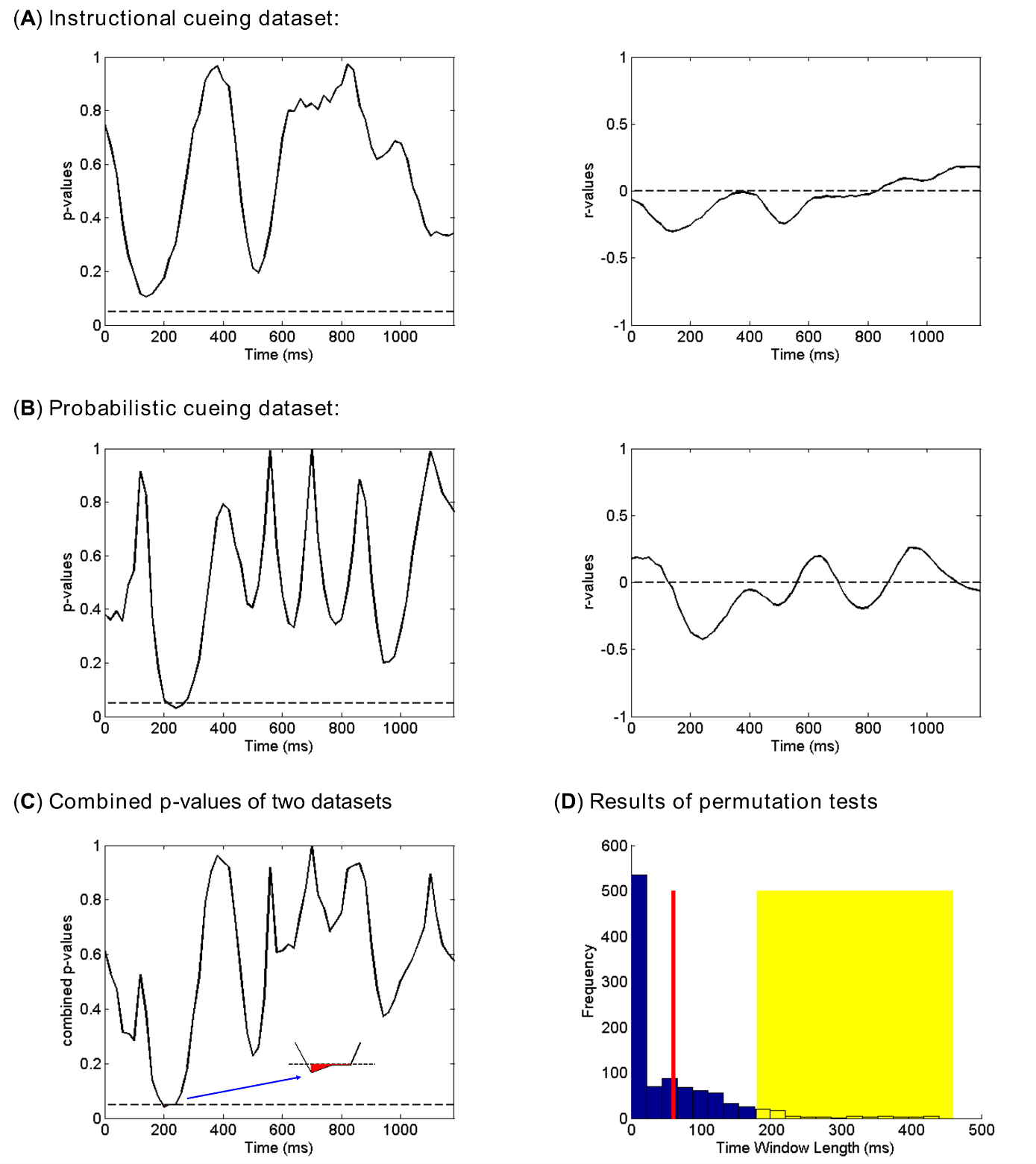


**Figure S5.** Results of between-subject correlation between cue-related alpha-based decoding accuracy and attentional modulation of target-related N1 in Instructional cueing dataset (*A*) and Probabilistic cueing dataset (*B*). The p values from the two datasets were combined by the Liptak-Stouffer meta-analysis (*C*). The *p* = 0.05 and *r* = 0 are indicated by the horizontal dash lines. Panel *D* shows the results of permutation tests for the two consecutive time windows identified in Panel *C*. The null distribution was estimated from 1000 permutations of the data, by randomly pairing one subject's decoding accuracy with another subject's N1 modulation. If the window length from the observed data (red line) falls within the top 5% of values from the null distribution (indicated by the yellow area), the observed window is considered to be significant.


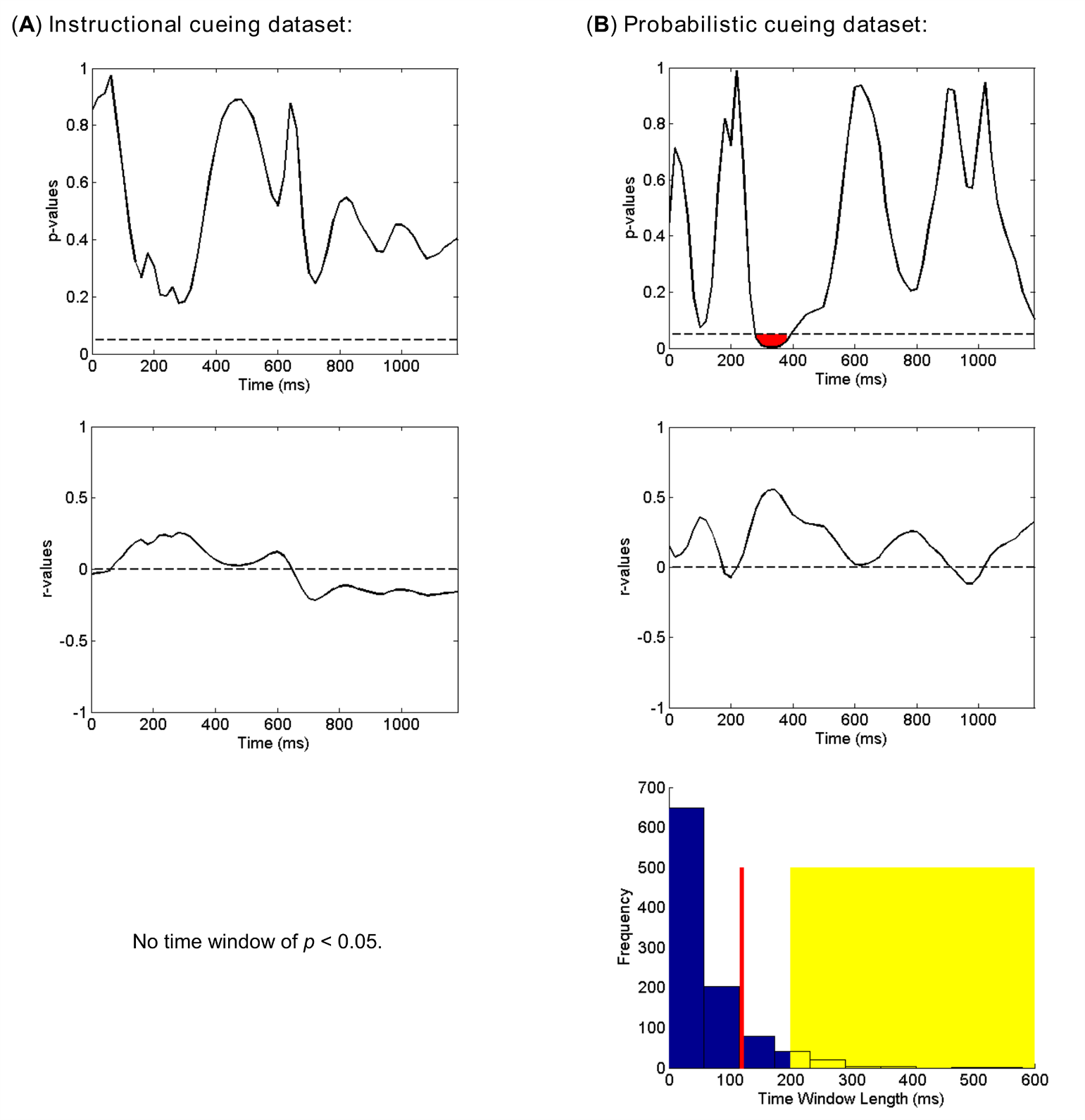


**Figure S6**. Results of between-subject correlation between cue-related ERP-based decoding accuracy and RT effects (*A*: RTs to attended targets; *B*: invalid RTs *minus* valid RTs) in Instructional cueing dataset (*A*) and Probabilistic cueing dataset (*B*). The *p* = 0.05 and *r* = 0 are indicated by the horizontal dash lines. For the dataset (*B*) with time window of *p* < 0.05, the null distribution was estimated from 1000 permutations of the data, by randomly pairing one subject's decoding accuracy with another subject's RT effect. If the window length from the observed data (red line) falls within the top 5% of values from the null distribution (indicated by the yellow area), the observed window is considered to be significant.


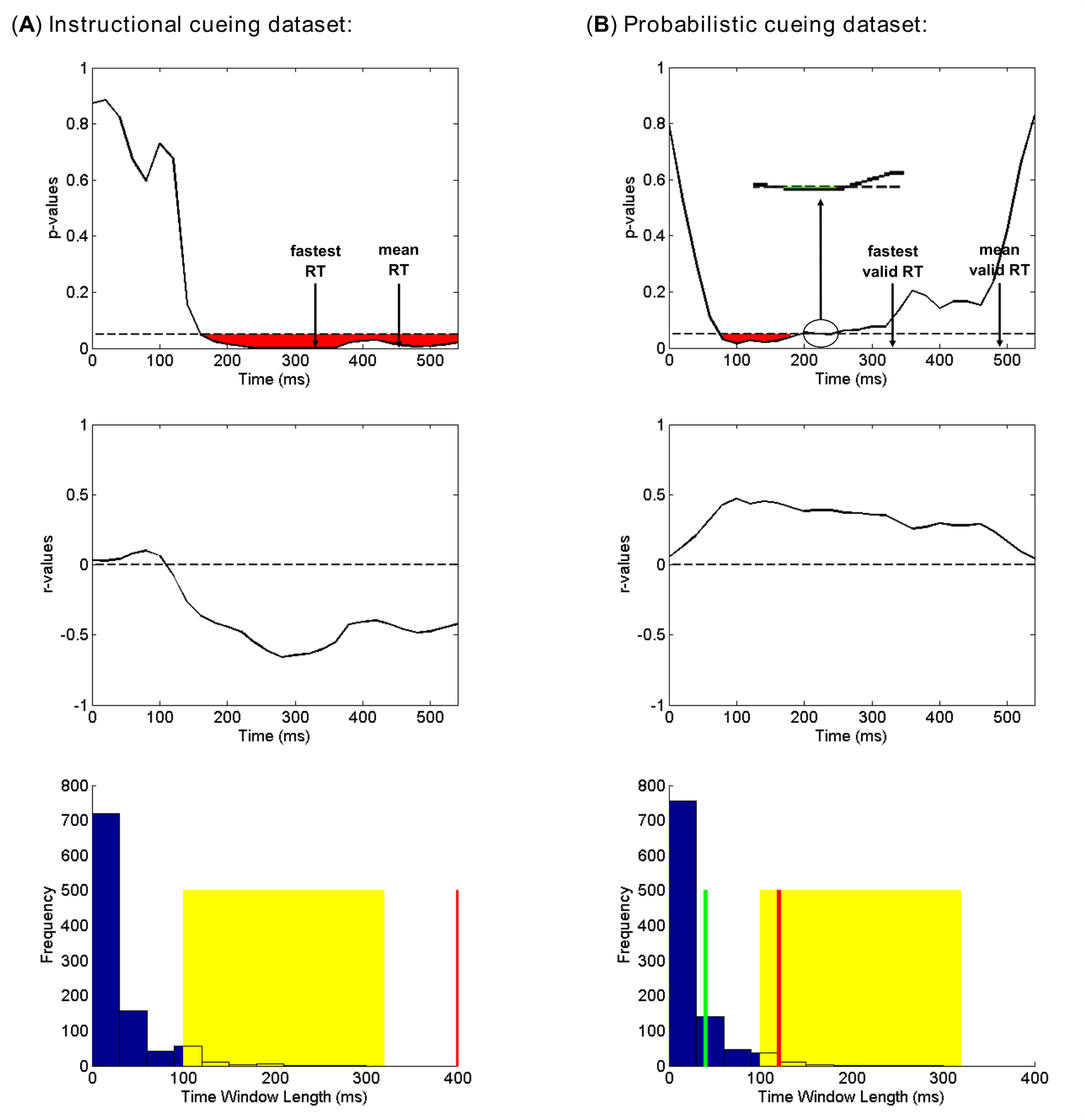


**Figure S7**. Results of between-subject correlation between target-related ERP-based decoding accuracy and RT effects (*A*: median RT to attended targets; *B*: median invalid RT *minus* median valid RT) in Instructional cueing dataset (*A*) and Probabilistic cueing dataset (*B*). The *p* = 0.05 and *r* = 0 are indicated by the horizontal dash lines. For each dataset, the null distribution was estimated from 1000 permutations of the data, by randomly pairing one subject's decoding accuracy with another subject's RT effect. If the window length from the observed data (red or green line) falls within the top 5% of values from the null distribution (indicated by the yellow area), the observed window is considered to be significant (red line in *A*: *p* < 0.001; red line in *B*: *p* = 0.033). The fastest (valid) RT refers to the fastest (valid) median RT across subjects, while the mean (valid) RT refers to the (valid) median RT averaged across subjects.

# References

Hong, X., Sun, J., Bengson, J. J., Mangun, G. R., & Tong, S. (2015). Normal aging selectively diminishes alpha lateralization in visual spatial attention. Neuroimage, 106, 353-363.

Rihs, T. A., Michel, C. M., & Thut, G. (2009). A bias for posterior alpha-band power suppression versus enhancement during shifting versus maintenance of spatial attention. Neuroimage, 44(1), 190-199.

Thut, G., Nietzel, A., Brandt, S.A., Pascual-Leone, A. (2006). Alpha-band electroencephalographic activity over occipital cortex indexes visuospatial attention bias and predicts visual target detection. J Neurosci, 26 (37), 9494–9502.

Worden, M. S., Foxe, J. J., Wang, N., & Simpson, G. V. (2000). Anticipatory biasing of visuospatial attention indexed by retinotopically specific alpha-band electroencephalography increases over occipital cortex. J Neurosci, 20(6), RC63.
